# Political economy of adolescent mental health and well-being globally

**DOI:** 10.1186/s12961-026-01477-2

**Published:** 2026-04-14

**Authors:** Olivia Biermann, Yusra Ribhi Shawar, Jeremy Shiffman, Seika L. Brown, Miranda Bain, Ismahan Shire, Sarah Baird, Pamela Y. Collins, Jennifer H. Requejo, Augustina Mensa-Kwao, Mark Tomlinson, Asha George, Manasi Kumar, Zeus Aranda, Rita Tamambang, Olayinka Omigbodun, Stefan Swartling Peterson, Mariam Claeson

**Affiliations:** 1 Department of Global Public Health, Karolinska Institutet, Tomtebodavägen 18 a, 17177 Solna, Stockholm, Sweden; 2 Bloomberg School of Public Health, Department of International Health, Johns Hopkins University, Baltimore, MD USA; 3 Paul H. Nitze School of Advanced International Studies, Johns Hopkins University, Washington, DC USA; 4 Youth-Led Global Research Initiative, Boston, USA; 5 Department of Global Health, George Washington University, Washington, DC USA; 6 Department of Mental Health, Johns Hopkins Bloomberg School of Public Health, Johns Hopkins University, Baltimore, MD USA; 7 Global Financing Facility, World Bank, Washington, DC USA; 8 Department of Global Health, Institute for Life Course Health Research, Stellenbosch University, Cape Town, South Africa; 9 School of Nursing and Midwifery, Queens University, Belfast, UK; 10 School of Public Health, University of the Western Cape, Cape Town, South Africa; 11 South African Medical Research Council, Cape Town, South Africa; 12 Department of Population Health, Institute for Excellence in Health Equity, New York University School of Medicine, New York, NY USA; 13 Partners In Health Mexico, Calle Primera Pte. Sur 25, Colonia Centro, 30370 Ángel Albino Corzo, Chiapas, Mexico; 14 Centre for Child and Adolescent Mental Health, College of Medicine, University of Ibadan, Ibadan, Nigeria; 15 Department of Psychiatry, College of Medicine, University of Ibadan, Ibadan, Nigeria; 16 School of Public Health, Makerere University, Kampala, Uganda; 17 Institute For Future Studies, Stockholm, Sweden

**Keywords:** Adolescent mental health and well-being, Political economy

## Abstract

**Background:**

The current generation of 1.3 billion adolescents (10–19-year-olds), most of whom live in low- and middle-income countries, face large and growing mental health problems. Collective action that could lead to significant improvement in adolescent mental health and well-being (AMH) remains limited. We analyse the factors shaping the global prioritization of AMH for funding and action and reflect on a way forward.

**Methods:**

We triangulate data from interviews with key informants knowledgeable in AMH; focus group discussions with youth representatives who are mental health advocates, some with lived experience of mental ill-health; and document review. We collected the qualitative data from 2021 to 2023, followed by thematic analysis and stakeholder consultations.

**Results:**

We identify four themes which shape the global prioritization of AMH. First, prevailing interpersonal and institutional stigma and discrimination directed against adolescents with mental health problems hamper attention to AMH. Second, limited data on the burden of mental health problems and evidence of what works have led to the perception among decision-makers that AMH is an intractable problem. Third, diverse ways of framing AMH are often viewed as a sign of weak alignment rather than as opportunities for coalition-building. Fourth, a wide variety and increasing number of stakeholders are involved in AMH, while the stakeholder landscape remains fragmented, inhibiting coalition-building for AMH.

**Conclusions:**

To overcome the barriers that currently impede the prioritization of AMH, we recommend that (1) stakeholders conduct an adolescent-led consultative process to develop an “umbrella framing”, supported by common metrics, (2) advocates use existing global platforms to shape the political priority for AMH, (3) decision-makers, funders and research partners invest in meaningful engagement of adolescents (with lived experience), researchers and implementing partners (4) identify a leadership, governance and accountability structure for a global coalition that could transform AMH and (5) conduct context-specific analyses to inform coalition-building nationally.

**Supplementary Information:**

The online version contains supplementary material available at 10.1186/s12961-026-01477-2.

## Background

Adolescents (10–19-year-olds) face increasing mental health problems. Mental health conditions represent 13% of the global burden of disease among adolescents and suicide was among the leading causes of death in this age group in 2019 [1]. Coronavirus disease 2019 (COVID-19) exacerbated the burden of mental health problems in adolescence, such as depression and anxiety [2].

Almost half of all mental disorders start during adolescence [3], often leading to long-term psychosocial challenges [4] and economic consequences [5]. The growing global evidence base has highlighted the need for multisector and multidisciplinary action to address the drivers of adolescent mental health and well-being (AMH). These include legal and regulatory interventions at the population level, education and support for caregivers, and environmental and psychosocial support at adolescent gathering spaces [6].

Funding for mental health, particularly AMH, is small compared with the magnitude of the problem [7]. In 2020, a global median of 2.1% of government health expenditure was allocated for mental health across age groups [8]. Development assistance for child and adolescent mental health during 2007–2015 constituted just 12.5% of total development assistance for mental health and 0.1% of development assistance for health [9]. Development assistance for AMH was largely spent on humanitarian emergencies, while 90 low- and middle-income countries received either no aid at all or less than 0.01 USD on average [9]. Philanthropic contributions constituted around 45% of total development assistance for mental health in 2015, while mental health received just 0.5% of all philanthropic health spending – the lowest proportion of any branch of health [10]. Although AMH is gaining financial interest [11], inadequate resources and budget allocation indicate the lack of priority. Addressing AMH effectively will require increased political commitment to the topic nationally and globally.

AMH has been increasingly recognized as a major public health issue. In 2020, 89 of 168 World Health Organization (WHO) Member States reported having an AMH plan [1]. AMH was the main topic of the 2024 Karolinska Institutet–United Nations Children’s Fund (UNICEF) conference on global child and adolescent mental health, the 2023 Global Forum for Adolescents and the 2021 UNICEF “The State of the World’s Children” report [12]. Mental health more broadly is included in the Sustainable Development Goals, while a WHO–World Bank initiative [13], the Lancet Commission on Global Mental Health and Sustainable Development [14] and the second Lancet Commission on Adolescent Health and Wellbeing [15] showcase examples of mental health integration into global finance and sustainable development. The 77th World Health Assembly 2024 approved a resolution to strengthen mental health and psychosocial support in emergency response. Yet, this increasing prioritization of the topic has not yet resulted in collective multisector and multidisciplinary action that could lead to transformational change. Previous analyses that explored the prioritization of global mental health (not AMH specifically) emphasized that the fragmented stakeholder community and the lack of global governance and a unified message have impeded advocacy efforts [16], whereas others emphasized the value of aligning AMH with other global development priorities [17].

Youth engagement in policy and programme processes is increasingly recognized as both a rights-based and instrumental strategy for change. Evidence from participatory governance and global policy frameworks indicates that youth engagement enables young people to influence agenda-setting, priorities and decision-making within multilateral institutions [18]. Youth engagement can also improve relevance and legitimacy in decision-making [15], even if youth advocates are not always more effective than other stakeholders [19] and it remains uncertain whether youth advocates actually drive political or policy change [20, 21]. “Meaningful engagement” refers to adolescents being involved early and continuously, rather than through a one-off consultation [22]. For example, at the agenda-setting stage, adolescents could help define which mental health issues deserve attention and their lived experiences can inform framing of the topic, as opposed to validation of adult priorities.

We analyse the factors shaping the global prioritization of AMH for funding and action and reflect on a way forward. As such, this work aims to support youth-driven strategies by key stakeholders to reduce fragmentation and mobilize resources in support of collective multisector and multidisciplinary action. Such action can help augment the global political priority of AMH and provide the impetus for sustained financing towards improved AMH outcomes.

## Methods

We triangulate data from interviews with key informants with experience and knowledge in AMH; focus group discussions (FGDs) with youth representatives who are mental health advocates, some with lived experience of mental ill-health; and document review. We collected the qualitative data from 2021 to 2023, followed by thematic analysis and stakeholder consultations.

### Conceptual underpinning

**Fig. 1:**
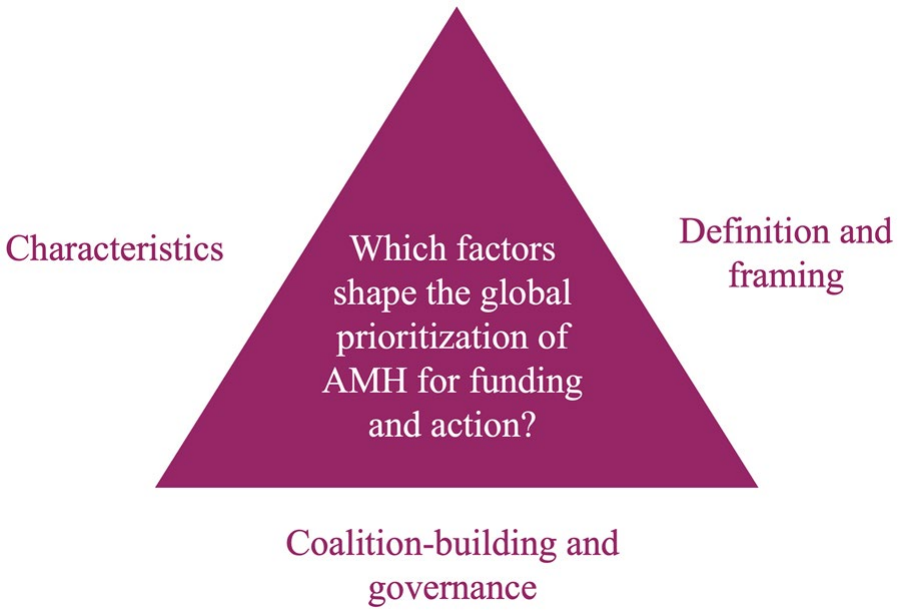
Framework for Political Economy Analysis of AMH adapted from Shiffman et al. [23]

Our analytical framework (Fig. 1) is based on work by Shiffman and colleagues [23, 24], whose frameworks draw on social science theory pertaining to collective action. The 2017 framework [23] identifies four challenges that global health networks face in generating attention and resources for the conditions that concern them: (1) *problem definition*: generating consensus on what the problem is and how it should be addressed; (2) *framing*: portraying the issue in ways that inspire external audiences to act; (3) *coalition-building*: forging alliances with these external actors, particularly ones outside the health sector; and (4) *governance*: establishing institutions to facilitate collective action. As in the 2007 framework [24], we considered the nature of the *political contexts* in which stakeholders operate, which strongly influence the level of political support. Moreover, we examined *issue characteristics* (for example, credible indicators, severity, effective interventions) [24], which influence the effectiveness of the stakeholders trying to address the issue.

### Document review

For the document review, O.B., M.B. and I.S. identified grey literature on AMH globally. The review did not aim to be comprehensive, but to identify key documents for triangulation with the findings from the interviews and FGDs. We used Google and hand-searched websites of United Nations (UN) and other international organisations, funders and governments who have AMH in their mandate or who have been vocal about AMH globally. We also considered documents suggested by the key informants or FGD participants. We applied key words such as “adolescent”, “adolescence”, “youth”, “young people” and “mental health”, searching for documents in English without any set timeframe. We identified 45 documents published in the 1989–2024 period, extracting data in the form of text passages on political context, characteristics, problem definition, framing, governance and coalition-building into Excel.

### Interviews, FGDs and consultations

#### Sample selection and recruitment of respondents

We used purposive and snowball sampling to identify a diverse set of key informants. Key informants were included if they had professional experience related to AMH at the global, regional and/or national levels; involvement in policy development, funding, research, advocacy or programme implementation relevant to AMH; and/or experience working within multilateral organisations, government agencies, academic institutions, civil society organisations or philanthropic bodies influencing AMH priorities. Individuals were excluded if they lacked relevant experience related to AMH or were unable to participate in an interview conducted in English. We compiled a list of key informants based on our knowledge of experts, asking colleagues in the field of AMH, and the respondents, for further recommendations. In total, we interviewed 26 key informants (10 female, 16 male). We did not use age or lived experience with mental ill-health as selection criteria. However, based on information provided during the interviews, the sample included at least four respondents under the age of 35 years and at least six with lived experience of mental ill-health.

We also used purposive and snowball sampling to identify youth representatives who are mental health advocates (aged 18–24 years) for the FGDs. S.L.B., a then 19-year-old AMH advocate, compiled an initial list of youth representatives based on her knowledge and global network. The list was complemented by input from the WHO Youth Council, the adolescent and youth constituency of the Partnership for Maternal, Newborn & Child Health (PMNCH) and citiesRISE. The snowball sampling led us to potential FGD participants up to 28 years of age, whom we included, as they had started advocating for AMH as adolescents and we were interested in their experience. We held four FGDs with a total of 17 respondents (10 female, 7 male). At least five respondents explicitly mentioned their own lived experience with mental ill-health during the FGDs.

To validate the findings from the qualitative data and the document review after completing the analysis, but prior to drafting the manuscript, we conducted four consultations. We employed convenience sampling, including existing groups of stakeholders, that is, an informal group of partners working on political economy analyses of AMH in Brazil, Kenya, Mexico, South Africa and Uganda, and the Commissioners and Youth Commissioners of the second Lancet Commission on Adolescent Health and Wellbeing. For the consultations with funders and youth representatives respectively, we contacted the individuals who had participated in the interviews and FGDs, including those who had declined participation previously. As such the consultations also served the purpose of member-checking. In total, 65 respondents (42 female, 23 male) took part in the consultations. Table 1 provides an overview of the respondents.

**Table 1:** Organisational affiliation, sex and country income level of respondents

	Female (*n*)			Male (*n*)		
	Interviewees	FGDs	Consultations	Interviewees	FGDs	Consultations
Total respondents	10	10	42	16	7	23
Organisational affiliation						
University	2	0	28	5	0	19
UN organisation	1	0	0	2	0	0
Non-governmental organisation	2	0	1	5	0	2
Funder	1	0	2	2	0	0
Advocate	4	0	1	1	0	0
Youth representatives (18–28 years of age)	0	10	10	1	7	2
Country income level						
Low-income country	2	0	0	0	0	1
Lower-middle-income country	3	7	8	4	5	4
Upper-middle-income country	1	2	14	4	0	7
High-income country	4	1	20	8	2	11
WHO region						
African Region	5	7	14	3	4	5
Region of the Americas	2	1	15	5	2	7
Eastern Mediterranean Region	0	0	0	1	0	0
European Region	2	0	5	3	0	5
South-East Asia Region	1	1	1	3	1	0
Western Pacific Region	0	1	7	1	0	6

#### Qualitative data collection and analysis

We collected the qualitative data, using semi-structured interviews and FGDs via Zoom. The topic guides (Annex 1) covered the different concepts mentioned in Fig. 1. After providing information about the study and obtaining written informed consent, we conducted the interviews and FGDs in English.

We analysed the data from interviews and FGDs using thematic analysis [25], using NVivo 11, triangulating the findings with data from the document review. We took the following analytical steps: (1) O.B. coded five interviews inductively, discussing the coding with Y.R.S. and M.C. (2) After coding all interviews, O.B. categorized the codes on the basis of the framework. (3) O.B., I.S. and M.C. integrated comparative and complementary information from the document review into the qualitative findings. (4) The findings were validated in the four consultation meetings held via Zoom. Any differences were resolved through discussion within the team. (5) O.B. and M.C. identified the main themes, primarily on the basis of data from key-informant interviews and FGDs, and drafted the manuscript with input from all co-authors.

## Results

**Fig. 2:**
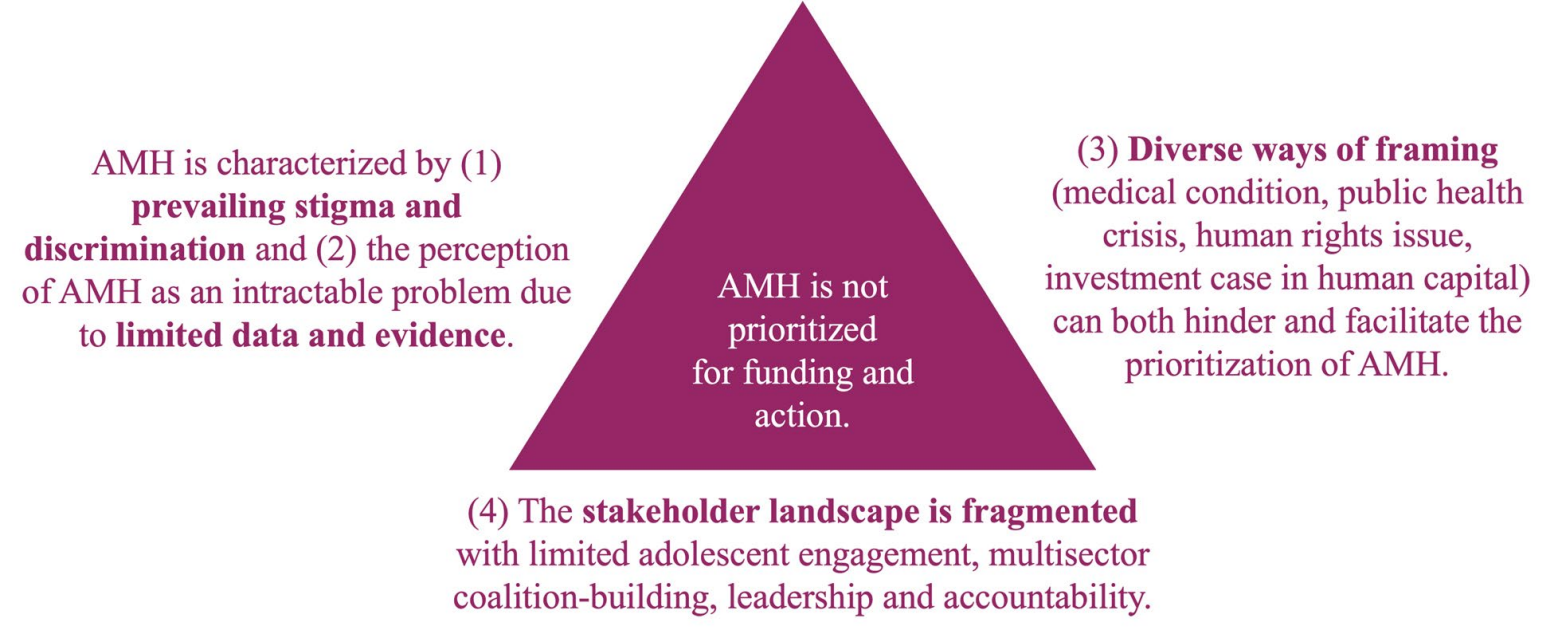
Summary of key findings on the political economy of AMH globally

We identified four themes shaping the global prioritization of AMH for funding and action. Figure 2 summarizes the key findings.

### Prevailing stigma and discrimination

Stigma […] sounds like a sort of simple thing, but it is a huge barrier […] especially for adolescents. [Interview 10, non-governmental organisation (NGO), female, lower-middle-income country]

Prevailing stigma and discrimination directed against adolescents with mental health problems are key factors – “the number one influence” (FGD 1) – hindering the prioritization of the topic, its funding and action. Public and interpersonal stigma (for example, by family members or politicians) and institutional stigma (for example, through policies and practices in schools) were highlighted as important. Adolescents with mental health issues may be hesitant to talk about mental health, as they may be “afraid to be seen as lunatics or people who cannot control their emotions or that have a substance use problem, are alcoholics, are drug addicts” (Interview 22, funding organisation, male, upper-middle-income country). These types of stigmata are in line with the findings of the Lancet Commission on Ending Stigma and Discrimination in Mental Health [26].

However, there was a sense that stigma and discrimination are diminishing, though unevenly and slowly. Adolescents described COVID-19 as a “wake-up call” (FGD 1), helping reduce stigma and discrimination and opening a window of opportunity to increase the political priority of AMH. Some respondents also stated that the pandemic led to heightened awareness of AMH, especially in high-income countries. Some respondents described how global developments (for example, climate change) and movements (for example, Black Lives Matter) have impacted the attention paid to AMH by providing entry points for related advocacy efforts.

The document review underlined that stigma and discrimination remain among the reasons why AMH continues to be neglected in policy and investment priorities [27, 28]. Stigma and discrimination are often seen as a barrier to policy change [26], for example, through lack of public demand for governmental action and investment.

### Limited data and evidence

It [AMH] is a huge problem […] There are so many fundamental [knowledge] gaps […] – the kind of like “wicked problem” area. (Interview 19, funding organisation, female, high-income country).

AMH has been characterized as an intractable problem mainly due to limited data that clearly demonstrate the burden and evidence of what works in prevention and treatment in adolescents, which has made decision-makers and donors hesitant to prioritize AMH. Some respondents emphasized that available studies are often limited to specific settings or vulnerabilities, such as school-based interventions for suicide prevention.

Reasons for limited data and evidence include difficulties in measuring AMH due to a lack of instruments and standardized indicators that can be adjusted to different contexts, some respondents stated. One respondent, an advocate from an upper-middle-income country, emphasized that researchers may be reluctant to focus on adolescents when additional efforts are required, such as parents’ consent. The document review furthermore highlighted the need to build better systems to capture AMH [1, 28]. Efforts to develop common metrics are underway, for example, by the WHO Global Action for the Measurement of Adolescent Health advisory group, the Adolescent Well-Being Initiative led by PMNCH and the UN, and the UNICEF Measurement of Mental Health among Adolescents and Young People at the Population Level.

We have to develop that culture of sharing information globally; sharing approaches of what works, what’s not working. […] Let us have that collective approach, lay out the key areas we need to focus on, set targets on the global level, break it down to regions and continents and then maybe, just maybe, we can win this battle. (FGD 3).

### Diverse ways of framing AMH

This space is being defined […]. Previously, mental health was known to be a business or a science for psychologists and psychiatrists, but now there are new players coming to this space […] opening up various understandings of mental health. (Interview 17, non-governmental organisation, male, lower-middle-income country).

The investment case in human capital – which emphasizes the socio-economic cost–benefits of investing in AMH – is the most widely employed framing of AMH according to respondents. This narrative can help inform government budget allocation, provide a rationale for allocative efficiency, justify expenditures and increase financial resources for AMH across sectors. Some of the reviewed documents [12, 29] and the first Lancet Commission on Adolescent Health and Wellbeing [30] put forward an investment case. Some respondents stated that this framing may risk reducing AMH to a solely transactional process. For example, one respondent from a university in a high-income country, stressed that adolescence is increasingly being recognized as a critical phase of life to invest in by those who have mainly future productivity, consumer potential or voting power in mind rather than improved AMH.

AMH is also commonly framed as medical conditions based on clinical diagnoses, which may be “the easiest answer, but not necessarily the best” narrative, since adolescents with mental health issues do not necessarily have or need a clinical diagnosis (Interview 4, university, male, upper-middle-income country). Some respondents explained the medical framing as problematic because it may perpetuate stigma linked to clinical diagnoses alone without addressing the social determinants. Respondents from universities mentioned anti-psychiatry movements that suggest that AMH should not be medicalized and that medicalizing AMH has created a marketing platform for the pharmaceutical industry to expand without improving AMH outcomes. The respondents argued for the importance of classifying, diagnosing and differentiating mental health conditions across a continuum to guide prevention and care [14].

When framing AMH from a public health perspective, respondents spoke about a “severe mental health crisis” (Interview 8, advocate, male, high-income country). Similarly, the 2021 US Surgeon General’s Advisory framed AMH as a public health crisis [31]. The public health narrative highlights how mental health problems can be a risk factor for other diseases and are interdependent with other public health crises such as gun violence and the epidemic of drugs use. This narrative was particularly raised by respondents from universities and NGOs as well as FGD participants. A variety of documents also employed the public health narrative emphasizing the importance of early interventions [12, 29, 32, 33].

Different framings may speak to different types of stakeholders and allow for new insights and understandings to emerge, the respondents stated. In contrast, we also found divergent perspectives which respondents said inhibited joint action across sectors: for example, AMH can be described as invisible versus a moral imperative, a topic requiring urgent action versus long-term structural reforms or as a wicked problem versus a complex but solvable topic. While certain narratives may draw attention to the topic, such as framing AMH as an investment case in human capital, the impact on the prioritization of AMH by decision-makers may still be questionable.

### Fragmented stakeholder landscape

I would say that the community per se is a little fragmented, but we are trying to come together now.” (Interview 18, UN organisation, female, low-income country).

While the AMH stakeholder landscape (Appendix) is expanding overall, the respondents specifically highlighted a growing funding landscape. Yet, adequate funding to change the trajectory of AMH remains unavailable across countries. Bilateral donors were perceived as powerful, investing in AMH in countries where it is not a priority and thereby potentially influencing policy change. Respondents expected increased financing of AMH to come from funders that have made explicit commitments to human development, adolescents, gender and/or youth empowerment such as the Bill and Melinda Gates Foundation. Reports have pointed out the fragmentation among funders and the importance of multilateral funding mechanisms and concessional financing [7], which are key to scaled and sustainable financing of AMH.

Stakeholders are increasingly aligned on the importance of AMH, but not necessarily regarding the causes and solutions, hampering joint action, as many respondents described. Stakeholders may point out different solutions for AMH reflecting their institutional mandates and justifying their institution’s presence in the field. At the same time, there was an understanding among the respondents that the “plurality of thought” is important “as long as it is in a framework where everyone agrees on the ultimate goal that they want to reach” (Interview 11, university, male, lower-middle-income country). The WHO and UNICEF Joint Programme of Work [34] was highlighted as an example of UN agencies aligning towards common AMH goals.

Adolescents, especially those with lived experience, were described as important stakeholders whose engagement in AMH is vital: “Our generation, GenZ, is really trying to talk about these issues […]. If there is any global or national agenda [on AMH] it is really started by young people.” (FGD 1). Examples of meaningful engagement include co-designing and testing AMH solutions, while barriers have been the legal age (often 18 years), as well as tokenistic engagement. Social media platforms have facilitated engagement, by educating adolescents about AMH, amplifying their voices and providing possibilities to connect with others and organise themselves as a movement, respondents stated. The importance of including young people was also emphasized in the document review [1, 35] and the Lancet Commission on Ending Stigma and Discrimination in Mental Health [26].

While there are many opportunities for AMH coalition-building, challenges remain. Many opportunities lie in schools, respondents stated. Adolescents emphasized, “Mental health has to be integrated in the education system; teachers and parents must also be included.” (FGD 3). Some respondents also mentioned opportunities for coalition-building between home care or protective care, religious institutions, youth detention centres and social protection services. Respondents from universities and NGOs as well as advocates elaborated on challenges to coalition-building, such as limited funding to support collaboration across sectors and the lack of evidence on the impact of multisectoral interventions. Stigma may hinder coalition-building, while coalition-building may help reduce stigma by broadening the perspectives on AMH, according to a respondent. The importance of coalition-building was also supported by the document review [6, 12, 27, 35].

To prioritize attention and investment in AMH, the importance of leadership was also recognized by most respondents and the document review [6]. Some respondents mentioned the need for leaders at local, national and global levels, and the important leadership roles of UNICEF, WHO, PMNCH, Orygen and United for Global Mental Health, governments, investors, adolescents and social media influencers. The engagement of influencers, such as singers, actors and athletes, has “started to change the conversation, particularly amongst young people in high-income countries. And that has then been adopted in many ways by politicians” (Interview 6, NGO, male, high-income country). A few respondents contested whether there is a need for a single leader, due to the many facets of AMH.

Different organisations and networks have been putting efforts into developing mechanisms to hold decision-makers to account for their commitments to AMH, respondents from NGOs and advocates stated. For example, PMNCH is developing a method for tracking progress on their global consensus statement related to adolescent and youth engagement and United for Global Mental Health has established a global independent monitoring platform “Countdown Global Mental Health 2030”, launched in 2021. The latter is the follow-up of a recommendation from the 2018 Lancet Commission on Global Mental Health and Sustainable Development.

What could change my mind about adolescent mental health being a global priority would be […] [that] we can actually easily track their commitments and actions that they are taking locally. […] I do not know who is going to be leading that charge. (Interview 14, NGO, male, lower-middle-income country).

## Discussion

This study identifies four interrelated factors shaping the global prioritization of AMH: stigma and discrimination, limited data and evidence, diverse ways of framing and a fragmented stakeholder landscape. These findings align with prior research highlighting stigma and discrimination as barriers to AMH prioritization [26–28], persistent evidence gaps in AMH [1, 14, 28] and fragmentation in AMH governance [6, 7, 12, 27, 34, 35]. However, this study furthermore emphasizes that prioritizing AMH will require addressing not only one but all barriers identified. In the following, we reflect on the way forward, focusing on the importance of strengthening AMH data and evidence, an “umbrella framing” for AMH, as well as a multisector coalition-building that could align stakeholders, reduce barriers and shape the global prioritization of AMH for funding and action.

Addressing gaps in AMH data and evidence requires targeted, system-level action and investment through a mix of public, multilateral, philanthropic and research funders. Priority areas include age- and sex-disaggregated AMH surveillance that clearly demonstrates the burden, expansion of implementation research co-designed with youth to assess what works in AMH prevention and treatment in diverse settings, and integration of AMH indicators into existing national and global health monitoring and accountability frameworks. Strengthening research capacity and data infrastructure is essential to guide resource allocation, evaluate scale-up of interventions, and ensure that increased political attention to AMH translates into effective and equitable services.

An “umbrella framing” for AMH, for example, a unifying statement, could help stakeholders embrace the diverse, complementary perspectives on AMH and tap into opportunities for coalition-building. First, an umbrella framing could underscore the need for multisector and multidisciplinary collective action, leveraging the different perspectives on AMH which our analysis illustrated. Second, an umbrella framing could recognize the many social determinants of AMH. Third, an umbrella framing could be supported by common metrics and standardized indicators to assess progress on AMH outcomes. A potential risk is the marginalization of certain stakeholders if the framing is not comprehensive or dominated by particular stakeholders. We recommend conducting an adolescent-led consultative process, with adolescents leading in youth-centred agenda-setting and problem-framing, while decision-making and implementation are shared with adult stakeholders. This approach aligns with Hart’s ladder of participation, distinguishing meaningful youth leadership from consultative engagement [27]. Such a process would be as important as the framing itself, drawing on the vast experience of key stakeholders.

International organisations and global platforms have key roles in coalition-building, resource mobilization and leadership for AMH globally. They must strengthen their collaboration, capitalizing on the convening power and mandates of UN organisations and the multi-constituency membership of other global platforms. They also have a role in strengthening the leadership of national governments and civil society, engaging adolescents and their families, funding organisations and other key stakeholders. One major adolescent-driven coalition-building effort was the 2023 Global Forum for Adolescents. We recommend identifying and using such windows of opportunity to shape the political priority for AMH, and investing in the meaningful engagement with adolescents, especially those with lived experience of mental ill-health.

AMH coalition-building could build on existing coalitions and networks. The Partnership for Global Mental Health has been proposed, but it does not focus on AMH as a major topic or adolescents as main actors. Our analysis suggests that a partnership may only impact AMH at scale if it is inclusive of adolescents and other AMH-specific stakeholders. We recommend conducting further analysis to identify a strong leadership, governance and accountability structure for a global coalition (of coalitions) that could transform AMH globally and nationally. We also recommend conducting context-specific analyses to inform coalition-building for AMH in countries.

## Strengths and limitations

This study’s strengths lie in the triangulation of data from various sources, the engagement of adolescents throughout the research process, the validation of findings through consultations and the involvement of a diverse and multidisciplinary team of co-authors. Limitations that may have led to the exclusion of important perspectives are an imbalance in the sampling (for example, the absence of voices of adolescents from low-income countries and those between 10–17 years of age and only a few adolescents with lived experience of mental health problems), the conduct of the data collection and consultations via Zoom instead of face-to-face and language being limited to English. Non-English-speaking participants were excluded due to resource and feasibility constraints related to translation, interpretation and transcription.

## Conclusions

To overcome the barriers that currently impede the prioritization of AMH, we recommend that (1) stakeholders conduct an adolescent-led consultative process to develop an “umbrella framing”, supported by common metrics; (2) advocates use existing global platforms to shape the political priority for AMH; (3) decision-makers, funders and research partners invest in meaningful engagement of adolescents (with lived experience); researchers and implementing partners (4) identify leadership, governance and accountability structures for global and national coalitions that could transform AMH; and (5) implement context-specific analyses to inform coalition-building nationally.

## Supplementary Information

Supplementary Material 1.

## Data Availability

The informed consent that all interviewees and focus group participants signed promises full anonymity. The key informants who participated in this study are well-known in the field of adolescent mental health and well-being in their countries and/or globally; making the full data set publicly available would breach their privacy.

## Appendix: Overview of key stakeholders (in alphabetical order), including examples

**Table:**

1	Academia and scientific groups and institutions (for example, Lancet Commission, African Population & Health Research Centre)
2	Adolescents (including those with lived experience of issues with their mental health and well-being), their families and communities
3	Advocates (for example, youth mental health advocates, influencers)
4	Funding organisations (for example, Bernard Family Foundation; Fondation Botnar; Global Fund for AIDS, TB and Malaria; Grand Challenges Canada; NIHR; Open Society Foundation; PEPFAR; The Children’s Investment Fund Foundation; Wellcome Trust)
5	Government institutions at national and sub-national levels (for example, ministries, agencies)
6	Inter-governmental organisations (for example, Organisation for Economic Co-operation and Development)
7	Local stakeholders (for example, volunteers, community-based organisations)
8	Media and social media platforms (for example, Facebook, Instagram, TikTok, Twitter, WhatsApp, YouTube)
9	Networks (for example, Alliance for Child and Adolescent Mental Health and Schools; Global Youth Action Network; Mental Health Innovation Network; Movement for Global Mental Health; TakingITGlobal; the Big 6 Youth Organisations (World Alliance of Young Men’s Christian Associations, World Young Women’s Christian Association, World Organization of the Scout Movement, World Association of Girl Guides and Girl Scouts, the International Federation of Red Cross and Red Crescent Societies and The Duke of Edinburgh’s International Award Foundation)
10	Non-governmental organisations (for example, citiesRISE; Generation Mental Health; Global Coalition for Youth Mental Health; Global Mental Health Peer Network; International Association for Child and Adolescent Psychiatry and Allied Professions; International Association for Youth Mental Health; the Partnership for Maternal, Newborn and Child Health, Plan International; Sangath; Save the Children; United for Global Mental Health; World Economic Forum)
11	Private sector (for example, pharmaceutical industry, insurance companies, Google, Microsoft) and other companies as part of their corporate social responsibility
12	Providers (for example, psychiatrists, psychologists, general practitioners, lay health workers, peer supporters)
13	Schools and the education system
14	Unions (for example, European Union, African Union)
15	United Nations organisations (for example, WHO, UNICEF, UNFPA, UNESCO, UNDP, UNHCR, UNAIDS, World Bank)
16	Other groups/providers working on mental health aspects of other issues (for example, SRHR, HIV, violence against women and girls, bullying, child marriage, menstrual stigma)

AIDS, acquired immune deficiency syndrome; HIV, human immunodeficiency virus; NIHR, National Institute for Health and Care Research; PEPFAR, US President’s Emergency Plan for AIDS Relief; SRHR, Sexual and Reproductive Health and Rights; TB, tuberculosis; UNAIDS, Joint United Nations Programme on HIV and AIDS; UNDP, United Nations Development Programme; UNESCO, United Nations Educational, Scientific and Cultural Organisation; UNFPA, United Nations Population Fund; UNHCR, United Nations High Commissioner for Refugees

## Abbreviations

AMH: Adolescent mental health and well-being
FGD: Focus group discussion
NGO: Non-governmental organisation
PMNCH: Partnership for Maternal, Newborn & Child Health
UNICEF: United Nations Children’s Fund
UN: United Nations
WHO: World Health Organization
