# Supplementary material for: Political economy of adolescent mental health and well-being globally
Source: Health Res Policy Syst. 2026 Apr 14;24:34. doi: 10.1186/s12961-026-01477-2 (PMC13081288; doi:10.1186/s12961-026-01477-2)
Supplement: Supplementary file 1 — Supplementary Material 1. [file 12961_2026_1477_MOESM1_ESM.docx]

**Political economy of adolescent mental health and well-being globally**

**Interview guide**

Broad introductory questions - level and development of priority

1. In your opinion, in how far is adolescent mental health and wellbeing a global political priority? What evidence would you cite to support your assertion?
2. How do you think has the global political priority for adolescent mental health and wellbeing changed over time?
   1. In the last decades?
   2. In the last 1-2 years?

Sociopolitical context

1. To what extend has the global social and political context influenced the political priority for adolescent mental health and wellbeing?
   1. For example, the Sustainable Development Goals?

Stakeholders worried about adolescent mental health and wellbeing

**I. Problem definition**

1. I use the term “adolescent mental health and wellbeing” during this interview, although I realize that you may use different words to describe this area.
2. Which terminology do you use?
3. How do you define the problem of adolescent mental health and wellbeing?
4. What is it about adolescent mental health and wellbeing that challenges or facilitates collective action to address it? (issue characteristics)
5. Who do you perceive as the main stakeholders engaged in adolescent mental health and wellbeing globally, i.e., individuals or organizations with an interest in the topic?
6. To what extent do you think stakeholders’ views are aligned or not when they speak about the problems and solutions regarding adolescent mental health and wellbeing?
   1. Where do they differ? Overlap?

**II. Framing/positioning**

1. When it comes to positioning adolescent mental health and wellbeing, how have stakeholders framed the problem for decision-makers (e.g., in governments, international and funding organizations)?
2. How well have stakeholders shown how serious the problem is?
3. How convincing are the solutions that stakeholders have proposed?
4. How effectively have stakeholders communicated solutions to attract political support?
5. From your point of view, how effective have stakeholders been in increasing the priority of adolescent mental health and wellbeing among decision-makers?
6. Do you have examples of stakeholders that have shifted priorities towards adolescent mental health and wellbeing?

**III. Governance and coalition-building**

1. From your perspective, how does the global community of stakeholders in adolescent mental health and wellbeing look like?
2. Which stakeholders make up this community?
3. To what extent do you think stakeholders at the grassroots (e.g., implementers) level are involved?
4. To what extent are adolescents involved?
5. In how far do you think the global community of stakeholders constitutes a unified community?
6. Have you encountered any stakeholders that oppose the prioritization of adolescent mental health and wellbeing?
7. Which stakeholders? Why?
8. How well has this opposition been handled?
9. In terms of leadership for adolescent mental health and wellbeing globally, is there any individual or organization who you consider a leader for adolescent mental health and wellbeing globally?
10. How has this leader been able to convene stakeholders to strengthen adolescent mental health and wellbeing worldwide?
11. What makes them an effective leader?
12. Thinking outside the field of adolescent mental health and wellbeing and the health sector, in how far have stakeholders forged alliances with those outside the field?

Additional questions

1. What is your view on the available data and research on adolescent mental health and wellbeing?
2. What do you think are the challenges in measuring adolescent mental health?
3. Who would you recommend I interview to learn more?
4. Would you have any additional comments?

**Focus group discussion guide**

**I. Problem definition**

1. While we use the term “adolescent mental health and wellbeing” during this focus group discussion, we wonder:
2. Which terminology do you use?
3. How do you describe the problems adolescents face regarding their mental health and wellbeing?
4. What is it about adolescent mental health and wellbeing that challenges or facilitates collective action to address it? (issue characteristics)

Level and development of priority

1. In how far is adolescent mental health and wellbeing a global political priority? What experience or evidence would you highlight that support your assertion?
2. How do you think has the global political priority for adolescent mental health and wellbeing changed over time?
   1. In the last decades?
   2. In the last 1-2 years?

Sociopolitical context

1. How have global social and political contexts influenced the priority for adolescent mental health and wellbeing?
   1. For example, the Sustainable Development Goals?

Stakeholders worried about adolescent mental health and wellbeing

1. Who comes to your mind the main stakeholders that are engaged in adolescent mental health and wellbeing globally, i.e., individuals or organizations with an interest in the topic?
2. To what extent do you think there is a global consensus among stakeholders around the problems and solutions regarding adolescent mental health and wellbeing?
   1. Where do they differ? Overlap?

**II. Framing/positioning**

1. How have stakeholders framed/positioned adolescent mental health and wellbeing for decision-makers (e.g., in governments, international and funding organizations)?
2. How well have stakeholders shown how serious the problem is?
3. Are you aware of the solutions that are being proposed by stakeholders?
4. Do you think the proposed solutions are convincing?
5. How effectively have stakeholders communicated solutions to attract political support?
6. From your point of view, how effective have stakeholders been in increasing the priority of adolescent mental health and wellbeing among decision-makers?
7. Do you have examples of stakeholders that have shifted priorities towards adolescent mental health and wellbeing?

**III. Governance and coalition-building**

1. From your perspective, how does the global community of stakeholders in adolescent mental health and wellbeing look like?
2. Which stakeholders make up this community?
3. To what extent do you think stakeholders at the grassroots (e.g., implementers) level are involved?
4. To what extent are adolescents involved?
5. In how far do you think the global community of stakeholders constitutes a unified community?
6. Have you encountered any stakeholders that oppose the prioritization of adolescent mental health and wellbeing?
7. Which stakeholders? Why?
8. How well has this opposition been handled?
9. In terms of leadership for adolescent mental health and wellbeing globally, is there any individual or organization who you consider a leader for adolescent mental health and wellbeing globally?
10. How has this leader been able to convene stakeholders to strengthen adolescent mental health and wellbeing worldwide?
11. What makes them an effective leader?
12. Thinking outside the field of adolescent mental health and wellbeing and the health sector, in how far have stakeholders forged alliances with those outside the field?

Additional questions

1. What is your view on the available data and research on adolescent mental health and wellbeing?
2. What do you think are the challenges in measuring adolescent mental health?
3. Who would you recommend I interview to learn more?
4. Would you have any additional comments?
